# Validation and comparison of instruments to identify frail patientes in primary care settings: Study protocol

**DOI:** 10.1186/s12913-016-1540-1

**Published:** 2016-08-05

**Authors:** Itziar Vergara, Francisco Rivas-Ruiz, Kalliopi Vrotsou, Eugenio Contreras-Fernández, Teresa Téllez-Santana, Mónica Machón, Ana Isabel Díez Ruiz, Yolanda de Mesa Berenguer, Andoni Bueno, Jazmina Núñez, M Carmen Saucedo Figueredo, Alonso Montiel-Luque, M Antonia Nava del Val, Raúl Quirós-López, Estefanía Carrasco, Gabor Abellan

**Affiliations:** 1 Unidad de Investigación APOSIs Gipuzkoa, Osakidetza, Donostia-San Sebastian, Spain; 2 Instituto Biodonostia, Donostia-San Sebastian, ᅟSpain; 3 Red de Investigación en Servicios de Salud en Enfermedades Crónicas, REDISSEC, Paseo Dr. Begiristain s/n; 2014, San Sebastián-Donostia, Spain; 4 Agencia Sanitaria Costa del Sol, Marbella, Málaga Spain; 5 Distrito de Atención Primaria Costa del Sol, Mijas, Málaga Spain; 6 Centro de Salud Beraun, OSI Donostialdea, Osakidetza, Renteria, Spainᅟ; 7 Unidad de Gestión Clínica de La Lobilla, Estepona, Málaga Spain; 8 Unidad Gestión Clínica Los Boliches, Fuengirola, Málaga Spain; 9 Unidad de Gestión Clínica San Miguel, Torremolinos, Málaga Spain; 10 Neuro-Oncology Group, Biodonostia Institute, Paseo Dr. Beguiristain s/n, San Sebastian, Spain; 11 Gérontopôle, Centre Hospitalier Universitaire de Toulouse, Toulouse, France; 12 INSERM UMR1027, Université de Toulouse III Paul Sabatier, Toulouse, France

**Keywords:** Frailty, Identification tools, Primary care

## Abstract

**Background:**

In the last few years several indices and tools, aimed at identifying frail subjects in various care settings have been developed. However, to date none of them has been incorporated into usual practice in the primary care setting. The purposes of this study are: 1) to evaluate the predictive capacity of the Tilburg Frailty Indicator (TFI), the Gérontopôle Frailty Screening Tool (GFST) and the KoS model together with two biomarker levels (SOX2 and p16INK4a) for adverse events related to frailty; 2) to determine differences in the use of healthcare services according to frailty.

**Methods/Design:**

Prospective multicentre cohort study with a 2-year follow-up. The study will be performed in primary care centres of Gipuzkoa and Costa del Sol, both located in Spain. Autonomous, non-institutionalized individuals aged 70 and over that agree to participate in this study will constitute the study population. A total of 900 individuals will be randomly selected from the healthcare administrative data bases of the participating health services. Data will be collected at baseline and at 1 and 2 years. The main independent variables assessed at baseline will be TFI outcomes, GFST and the KoS model, together with the expression of SOX2 and p16INK4a levels. During follow-up, loss of autonomy, the occurrence of death and consumption of healthcare resources will be assessed.

**Discussion:**

The main focus of this work is the identification and evaluation of several instruments constructed under different rationales to identify frail subjects in primary care settings. The resulting outcomes have potential for direct application to the primary care practice. Early identification of the onset of functional impairment of elderly is an essential, still unresolved aspect in the prevention of dependence in the scope of primary care.

## Background

Ageing is accompanied by a series of physiological changes which lead to a gradual loss of adaptation to the demands of the environment and increased vulnerability. The most severe expression of ageing is the clinical condition of frailty [1]. This is defined by the gradual reduction in resilience and reserve capacities leading to an overall deterioration in health. This deterioration can subsequently progress to dependence, intensive use of healthcare resources, and death [2, 3]. Frailty is a powerful indicator of the state of health of the elderly [3] but progress needs to be made to understand the process of frailty, its onset, its determining factors and consequences [4].

In the last few years several indices and tools aimed at identifying frail subjects in various care settings have been developed. However, to date none of them has been incorporated into usual practice in a primary care setting.

### Existing instruments

Instruments for the identification of frail individuals are based on different approaches and rationales [5–7]. Three of them are considered in this study.

1. *Instruments based on rules:* their construction is based on multiple regression analysis models. This group includes, among others: a) The frailty phenotype model proposed by Fried in 2001, that evaluates the presence of five criteria: slow walking speed, reduced grip strength, low physical activity, exhaustion and unintentional weight loss [2], b) The Tilburg Frailty Indicator (TFI), that has been considered [5, 8] as the most suitable to be used in primary care due to its simplicity and psychometric characteristics [9], and c) The KoS model, a new model developed by this investigation group, which considers the age of the subject, the presence of polipharmacy and the Timed Up and Go (TUG) test results [10].
2. *Instruments based on clinical judgement:* these instruments are designed to classify individuals based on the clinical history and the clinician’s knowledge and assessment. Two instruments are included in this group: a) the Clinical Frailty Scale (CFS) [11], from the Canadian Study of Health and Ageing (CSHA) group, and b) the Gérontopôle Frailty Screening Tool (GFST) [12].
3. *Frailty biomarkers:* a total of 63 different possible frailty biomarkers (genes) have been suggested. Most of these genes are involved in oxidative stress, inflammation and metabolism although there is no clear consensus on their validity [13, 14]. Interleukin 6 (IL-6) is probably the most standardised marker for this purpose [13]. However, it is of major interest to explore new frailty markers. It is well-known that ageing is associated with a reduction in the regenerative tissue properties. Given that the regenerative capacity of tissues relies on stem cells, ageing and frailty may be at least partially, a consequence of a disorder in stem cell regulation [15]. The transcription factor SOX2 (denominated from Sex determining region Y-related HMG box2) is very important in the biology of stem cells. Also it is one of the 4 factors necessary to re-programme differentiated cells in induced pluripotent stem cells [16], to ensure capacity for self-renovation of this cell population [16–20] and regulate their de-differentiated state [16–18]. Surprisingly, its function in tissue and in the ageing body still needs to be investigated. Interestingly, it is known that its expression reduces in different brain areas with ageing [19]. These results indicate that reduced SOX2 levels may be a marker of the ageing process and is suggested as a possible biomarker of frailty. Additionally, the tumour suppressor p16INK4a, which is an inhibitor of the cell cycle progression and an important mediator of cellular senescence has been postulated as a biomarker of ageing [21, 22] and could also represent an indicator of frailty.

### Healthcare resources

Frail subjects present a high rate of adverse events such as dependence, institutionalisation or death. In the Fried 2001 study the prevalence of hospitalisation of frail patients was 59 % in the first year compared to 33 % in the non-frail population [2]. These data lead to the hypothesis that, in our setting, frailty may be associated with a different pattern of health resources consumption. Frail individuals are expected to do a more frequent and intensive use of the available healthcare resources.

The goal of this project is to evaluate the predictive capacity of the TFI, GFST, KoS model, as well as of the SOX2 and p16INK4a levels for adverse events related to frailty. An additional objective is to describe the existing differences in the pattern of healthcare services use according to frailty status.

## Methods/Design

### Design

Prospective multicentre cohort study with a two-year follow-up of autonomous and community dwelling subjects aged 70 and over*.*

### Scope

The study will be performed in the primary care areas of Gipuzkoa and Costa del Sol, in the north and south shores of Spain. At these locations, primary care services provide care to 384,683 and 473,478 users of whom 11 % are aged 70 and over.

### Study population

Autonomous (Barthel test >90 points), not institutionalised individuals (living at their home or with a family member) aged 70 and over, who agree to participate in the study. Subjects in a terminal situation defined according to the guidelines of the Spanish Society of Palliative Care [23], those who reside more than 6 months per year in a different area and those with difficulty communicating in Spanish or Basque (in Gipuzkoa area only), will be excluded from the study. Patients will be randomly selected from the administrative databases of the participating healthcare services. The sample will be representative of the populations of interest in terms of age and sex.

**Fig. 1:**
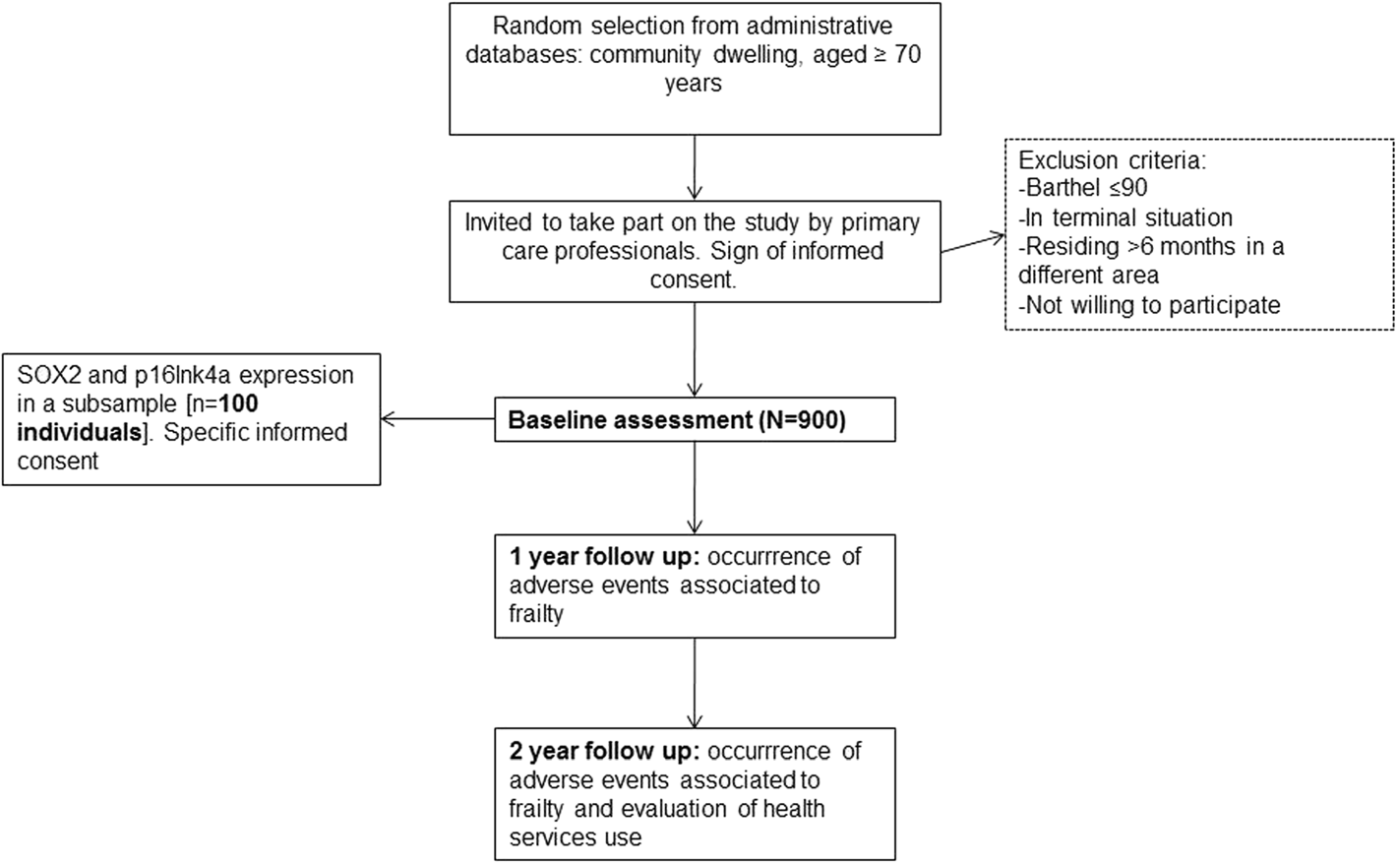
Study protocol description

Essential socio-demographic information related to all patients who comply with the selection criteria will be collected with the purpose of comparing subjects who finally participate to those who refuse or abandon. A detailed overview of the planned flow of participants is provided in Fig. 1.

### Variables

The variables collected throughout the study are presented in Table 1.

**Table 1:** Study variables

Domain	Variables or Questionnaires	Description	Assessment
Socio-demographic	Date of birth, health centre, sex		Baseline
Control Data	Date of interview, person interviewed		Baseline
Frailty	Modified Fried frailty criteria	Unintentional weight loss, low level of physical activity, low energy/tiredness, muscle weakness, slow movements	Baseline
	Tilburg Frailty Index	15 items, 3 domains (physical, psychological and social)	Baseline
	KoS Model	Age + Polypharmacy + Timed Up-and-Go test	Baseline
	Gérontopôle Frailty Screening Tool	6 items + Clinical judgement	Baseline
Health-related lifestyle	Physical activity - Short Physical Performance Battery	3 tests: balance, walking speed and getting up and sitting down from a chair 5 times	Baseline
	Tobacco use	3 items	Baseline
	Malnutrition screening - Mini Nutritional Assessment	18 items	Baseline
Health Status	Self-perceived health	1 item	Baseline
	Healt-related quality of life - EuroQol 5D	5 items + 0–100 visual analogue scale	Baseline
	Cognitive state - Mini-cog test	14 items, 5 domains: orientation, attention, concentration, calculation, memory, language and construction	Baseline
	Geriatric syndromes	Sight, hearing and falls	Baseline
	Comorbidity (I)	Review of 10 diseases in clinical record	Baseline
	Comorbidity (II): Cumulative Illness Rating Scale	Review of 19 diseases in clinical record	Baseline
	Comorbidity (III): Charlson Comorbidity Index	Review of 13 diseases in clinical record	Baseline
	Drug use	Assessment of 5 therapeutic groups of drugs	Baseline
Functional capacity	Basic activities of daily living - Barthel Index	10 items	Baseline, 1 year, 2 years
	Instrumental activities of daily living - Lawton & Brody scale	8 items	Baseline
Biomarkers	2 markers	SOX2 and p16lNK4a	Baseline
Health resources use	Use of Primary and Specialty Healthcare Resources	Primary Care, ER, Hospitalization	1 year, 2 years
Adverse events	Loss of autonomy; Death		1 year, 2 years

#### Outcome variables

1. The occurrence of adverse events related to frailty, mainly loss of autonomy, defined as Barthel ≤90 [24]. Death is considered to be an adverse event associated with frailty, but it is not planned to be studied as a single outcome as its expected frequency would not allow it (details in sample size section). Loss of autonomy and death will be studied jointly during a secondary analysis phase.
2. The consumption of healthcare resources considering the number of primary care consultations by medical and nursing staff, the number of visits to the emergency department, number of specialised consultations, number of hospital admissions and days of stay, number of admissions in short stay departments.

#### Main independent variables

- *The Tilburg Frailty Index (TFI)* is a self-administered questionnaire that takes 14 min to fill in. It contains 15 items split into three components: physical, psychological and social. It collects information on the degree of autonomy, cognitive capacity, mood, physical functionality and social network support. Its total score ranges from 0 to 15 points. This questionnaire is being translated and validated in Spanish by the present investigation group.
- *The Gérontopôle Frailty Screening Tool (GFST)* is designed to be administered to people aged 65 and over, autonomous, without concurrent acute clinical pathology. It is comprised of an initial questionnaire guiding the doctor to assess some general signs or symptoms that could suggest the presence of an unidentified frailty situation. In a second section the professional is asked to express their clinical opinion on the existence of frailty. The final GFST tool result is based on this very clinical opinion.
- *The KoS model* is comprised of the individual’s age at the time of the interview, the presence of polipharmacy, considered as the consumption of four or more prescribed medicines and the result of the TUG test [25]. The KoS model (publication under review) presents an area under the curve (AUC) of 0.822.
- *The expression of SOX2 and p16INK4a* will be measured in a blood obtained from a subsample of patients. First, these samples will undergo an RNA purification by means of the RNeasy Kit (Qiagen). The RNA will be retro transcribed using the reverse transcription cDNA High Capacity kit of Applied Biosystems. Then, the expressions of SOX2 and p16INK4a will be determined by means of quantitative PCR (qPCR) using specific primers or probes and the equipment ABI Prism® SDS 7300 Real Time PCR System from Applied Biosystems. Expression levels will be standardised with those for expression of the enzyme Glyceraldehyde-3-Phosphate Dehydrogenase (GAPDH) and compared to the IL-6 levels which will be studied in parallel.

#### Secondary independent variables

- *Socio-demographic data:* Date of birth, sex, level of studies, income level and place of residence.
- *Healthcare-related lifestyle:* 1) Physical activity measured with the Short Physical Performance Battery (SPPB), which includes evaluation of balance, gait speed and getting up and sitting in a chair [26]. 2) Tobacco consumption: three items have been included to establish smoking habits according to the WHO [27]. 3) Screening for malnutrition, evaluated with the Mini Nutritional Assessment test [28].
- *State of health:* 1) Self-perceived health measured with a single item: “Overall, you would say your health is…” with five response options: excellent, very good, good, fair, and poor, 2) Health-related quality of life evaluated using the EQ-5D questionnaire (EuroQuol Group) [29], 3) Cognitive status with the Mini Mental State Examination (MMSE) [30], 4) Existence of geriatric syndromes: visual and auditory deficits, the occurrence of falls during the year prior to inclusion, 5) Comorbidity measured by the Cumulative Illness Rating Scale [31] and the Charlson Comorbidity Index [32] and 6) Prescription drugs. This information will be collected by means of personal interviews and from the clinical records.
- *Functional autonomy and capacity:* reflects the capacity to perform basic and instrumental activities of daily living. The Barthel Index will be used to evaluate basic activities and the Lawton and Brody scale will be used to assess instrumental ones [33].

### Sample size

The experience of this investigation group with similar data, leads to the hypothesis that at the end of follow-up 15 to 20 % of the recruited subjects will have become dependent according to the defined criteria. Thus, for a total of 650 subjects it is calculated that 100 to 130 will present this event. Following the rule of 10 events per variable for the logistic regression models, the expected distribution in the two outcome groups (the one presenting adverse events related to frailty and the group not presenting them), will allow to construct logistic regression models considering several indices at the same time, in case a simultaneous effect is suggested when predicting the adverse event. It will also enable studying the predictive capacity of categorised indices which generally increase the requirements of the total N. This N should also allow for possible subgroup analyses if so indicated by the data.

It is estimated that death will occur in a much lower percentage, not more than 5 %. Because of its expected low-frequency, this result will not be studied individually. In addition, it is expected that approximately 20 % of the recruited subjects will not attend the arranged appointment with the study personnel or will not provide informed consent, and therefore will have to be excluded from the study. Similarly, another 20 %, will not provide data at the end of the study for various other reasons. Considering these follow-up losses the initial sample size has to be increased by 40 %, recruiting a total of 900 subjects (450 in each setting).

Genetic analyses will be performed in a subsample of 100 subjects (50 in each setting) randomly selected considering their frailty status measured by the TFI. This approach is used to ensure the feasibility of the project given the level of resources required to perform the genetic expression analyses proposed.

### Data management

All selected subjects will be contacted by letter and phone and will receive information about the project. Those interested in participating will undergo the Barthel test. Subjects with a Barthel ≤90 will be excluded. Basic socio-demographic data of subjects who decline participation will be collected and analysed with the purpose of identifying possible selection or participation bias.

Those interested will be informed in detail about the study; will sign an informed consent and will undertake a baseline assessment performed by a trained nurse. Blood samples will be extracted in the selected subsample. Information on current clinical diagnoses and consumption of medicines will be obtained from the clinical records.

The variables related to the use of healthcare resources will be obtained from the electronic clinical records and the administrative databases of the healthcare services. Autonomy status will be assessed with the Barthel test at 1 and 2 years through a telephone interview. The event of death will be checked on clinical records.

### Statistical analysis plan

The study unit is the patient. Categorical variables will be described with frequencies and percentages and continuous variables with means and standard deviations (SD) or medians and interquartile ranges (Q1, Q3), according to their degree of symmetry. Categorical variables will be compared with the chi-square or Fisher’s exact test. Continuous variables with a Normal distribution will be compared with the Student’s *t*-test and the non-parametric Wilcoxon rank-sum test will be implemented for variables with distributions other than Normal. The following statistical analysis plan will be performed:

1. *Association between different indices:* The relationship between the scores of different indices as well as with biomarkers’ levels will be examined with the Pearson’s and Spearman’s correlation coefficients. Point estimates of these correlations and their 95 % confidence intervals (95 % CI) will be provided. Concordance between the tests after categorising patients according to cut-off points established in the scientific literature will also be examined. This concordance will be studied with the Kappa coefficient.
2. *Outcome of dependence during follow-up:* The relationship of all implemented indices with dependence (yes/no) at the end of the study will be verified. This relationship will be studied by means of binary logistic regression models. In any case, the predictive capacity of the indices will be verified considering them both as continuous and as categorical variables, according to published references when available. In case there are no publications (e.g. SOX2) possible cut-off points will suggested by means of ROC curves. During this analysis phase the external validation of the KoS model, applied to this cohort of subjects, will also be examined. The predictive effect of each index, both univariate and multivariate will be studied. The aim is to study whether the simultaneous consideration of more than one of the included indices can improve the prediction of this adverse event and also quantify this improvement in terms of AUC, sensitivity and specificity of the derived probabilities.
3. *Differences in terms of resources consumption by frail and non-frail subjects:* Frequency of consumption as well as duration of possible hospital admissions will be compared between the two groups. These data usually follow skewed distributions therefore comparisons will most likely be performed by means of median differences. Confidence intervals will be estimated and non-parametric statistics will be used in these cases.

All statistical analyses will be performed with the SAS 9.3 and the R 3.1.0 software.

## Discussion

The priority of frailty in the context of research in healthcare services is highlighted, among others, by the European Commission report on ageing (2012) which specifies that *“the reduction of disability and dependence by means of appropriate measures in the frailty process should be at the forefront of innovation in all healthcare policies”* [34].

This project is aimed to increase the evidence of suitable instruments able to identify frail subjects in primary care settings and to study the use of healthcare services of these subjects. The identification of frail subjects is highly relevant because it can help to design interventions adapted to their needs, which in turn could help stopping or at least delaying the natural advance towards dependence. Primary care is the most appropriate setting to identify frail individuals because of its proximity and accessibility [35]. In this healthcare setting, the identification of frailty should be simple and require little time [36].

The main limitation of this work is related to the possibility of having a representative sample of the non-dependent elderly population in our setting given the tendency of subjects with better heath to be more likely to participate. This can limit the incidence of adverse events sought during follow-up. For this reason, a sample of advance age was selected in which the likelihood of occurrence of these events is greater.

Another limitation is the expected rate of losses to follow-up. To reduce these losses, contact data will be verified and additional contacts (carer, spouse or descendants) will be requested in order to maintain close contact with the participants.

The main focus of this study is the evaluation of instruments and strategies for identifying frail subjects, a goal that has major potential for direct application on primary care settings. Early identification of the onset of functional impairment in elderly subjects is an essential but still unresolved aspect in the prevention of dependence. The final goal of this study is to offer to the scientific community a suitable instrument to identify frail individuals in the primary care setting.

## Abbreviations

CFS, Clinical Frailty Scale; CSHA, Canadian Study of health and Aging; EuroQuol Group, EQ-5D questionnaire; GFST, Gérontopôle Frailty Screening Tool; IL-6, Interleukin 6; MMSE, Mini Mental State Examination; SD, standard deviations; SOX2, (denominated from Sex determining region Y-related HMG box2); SPPB, the Short Physical Performance Battery; TFI, Tilburg Frailty Indicator; TUG, Timed Up and Go test; WHO, World Health Organization
